# Impact of the gene polymorphisms in the renin-angiotensin system on cardiomyopathy risk: A meta-analysis

**DOI:** 10.1371/journal.pone.0295626

**Published:** 2024-01-02

**Authors:** Xiaoxiao Jia, Liping Meng, Weiliang Tang, Liping Sun, Fang Peng, Peng Zhang

**Affiliations:** 1 Department of Pathology, Shaoxing People’s Hospital, Shaoxing, China; 2 Department of Cardiology, Shaoxing People’s Hospital, Shaoxing, China; Mansoura University Faculty of Medicine, EGYPT

## Abstract

Due to the inconsistent findings from various studies, the role of gene polymorphisms in the renin-angiotensin system in influencing the development of cardiomyopathy remains unclear. In this study, we conducted a systematic review and meta-analysis to summarize the findings regarding the impact of angiotensin converting enzyme (ACE) I/D, angiotensinogen (AGT) M235T, and angiotensin II Type 1 receptor (AGTR1) A1166C gene polymorphisms in patients with cardiomyopathy. We performed a comprehensive search of several electronic databases, including PubMed, Embase, the Cochrane Library, and Web of Science, covering articles published from the time of database creation to April 17, 2023. Studies on the assessment of genetic polymorphisms in genes related to the renin-angiotensin system in relation to cardiomyopathy were included. The primary outcome was cardiomyopathy. Risk of bias was assessed using the Newcastle-Ottawa Scale scale. The meta-analysis includes 19 studies with 4,052 cases and 5,592 controls. The ACE I/D polymorphisms were found to be associated with cardiomyopathy (allelic model D vs I: OR = 1.29, 95CI% = 1.08–1.52; dominant model DD+ID vs II: OR = 1.43, 95CI% = 1.01–2.02; recessive model DD vs ID+II: OR = 0.79, 95CI% = 0.64–0.98). AGT M235T polymorphism and cardiomyopathy were not significantly correlated (allelic model T vs M: OR = 1.26, 95CI% = 0.96–1.66; dominant model TT+MT vs MM: OR = 1.30, 95CI% = 0.98–1.73; recessive model TT vs MT+MM: OR = 0.63, 95CI% = 0.37–1.07). AGTR1 polymorphism and cardiomyopathy were not significantly associated under allelic model A vs C (OR = 0.69, 95CI% = 0.46–1.03) and recessive model AA vs CA+CC (OR = 0.89, 95CI% = 0.34–2.30), but under the dominant model AA+CA vs CC (OR = 0.51, 95CI% = 0.38–0.68). The current meta-analysis reveals that polymorphisms in ACE I/D may be a genetic risk factor for cardiomyopathy. There is an association between AGTR1 gene polymorphisms and risk of cardiomyopathy under the specific model.

## Introduction

Cardiomyopathies are heart disorders characterized by structural and functional abnormalities. They include dilated cardiomyopathy (DCM), hypertrophic cardiomyopathy (HCM), arrhythmogenic right ventricular cardiomyopathy (ARVC), restrictive cardiomyopathy (RCM), and unclassified cardiomyopathy [1]. Among these, HCM is the most common genetic heart disease, characterized by left ventricular hypertrophy, which can lead to heart failure and sudden cardiac death. The prevalence of HCM in the general population ranges from 1 in 200 to 1 in 500 for asymptomatic cases and 1 in 3000 for symptomatic cases [2,3]. DCM, on the other hand, is a significant genetic heart disease characterized by left ventricular dilation and systolic dysfunction. It is one of the leading causes of heart failure and may require cardiac transplantation in severe cases. The estimated prevalence of DCM in the general population is 1 in 2500 [4].

Over the past three decades, the recognition of disease-causing genetic variants and advancements in understanding the genetic basis of cardiomyopathies have highlighted the importance of genetic factors in their pathogenesis [5–7]. Among these factors, the renin-angiotensin system (RAS) plays a crucial role. The RAS is involved in volume homeostasis and exerts powerful effects on the heart and circulatory systems. In patients with cardiovascular disease, RAS activity is often increased [8]. Several reports have indicated that gene polymorphisms in the RAS contribute to the development of cardiomyopathy [9]. Key components of the RAS include angiotensin converting enzyme (ACE), angiotensinogen (AGT), and angiotensin II Type 1 receptor (AGTR1).

The ACE gene, located on chromosome 17q23, exhibits a genetic polymorphism in intron 16 characterized by the presence of an insertion (I) or deletion (D) of a non-coding base pair Alu repeat sequence [10]. Several studies reported that ACE I/D polymorphism is associated with Cardiomyopathy [11–13]. AGT, released from the liver, is cleaved by renin. The AGT M235T gene polymorphism, denoting a MET to Thr substitution at codon 235, has been associated with elevated AGT levels. Individuals with the 235TT genotype exhibit 10% to 20% higher plasma AGT levels compared to those with the 235MM genotype [14]. The genotype of AGT may serve as a potential risk factor as a positive association has been reported between the AGT M235T polymorphism and HCM [15]. The AGTR1 gene, located on chromosomes 3q21–3q25, consists of four introns and five exons, resulting in four distinct alternatively spliced transcripts. More than 20 polymorphisms have been identified in this gene, with the 1166A>C polymorphism in the 3’ untranslated region being the most extensively studied in the literature [16]. Similarly, linkages between AGTR1 A1166C and cardiomyopathy has been reported [17–19].

Despite the studies linking the RAS to cardiomyopathy, the findings from different studies have been contradictory [20–22], and the role of the gene polymorphisms in the RAS system remains unclear in influencing the development of cardiomyopathy. Given the inconsistent results from different studies, we conducted a systematic review and meta-analysis to summarize the findings regarding the role of ACE I/D, AGT M235T, and AGTR1 A1166C gene polymorphisms in the RAS in patients with cardiomyopathy.

## Materials and methods

This study followed the Preferred Reporting Items for a Systematic Review and Meta-analysis (PRISMA) statement.

### Search strategy

We searched PubMed, Embase, the Cochrane Library, and Web of Science for case-control studies assessing the association of genetic polymorphisms in RAS-related genes (ACE I/D, AGT M235T, and AGTR1 A1166C) with cardiomyopathy, with a time limit of from database creation to April 17, 2023. Cardiomyopathy, RAS-associated genes, gene polymorphisms, and case-controls were searched individually or in combination, and English was the only language allowed. Only human studies were included, and the search strategy is detailed in the S1 Table.

### Study selection

Inclusion criteria were as follows: (1) studies that included patients with cardiomyopathy; (2) studies that investigated any gene of ACE I/D, AGT M235T, or AGTR1 A1166C; (3) study design should be a case-control study; and (4) studies that were published in English. Exclusion criteria were (1) abstracts, letters, conference talks, or reviews; (2) studies that involved patients with non-cardiomyopathy; (3) duplicate studies that provided overlapping data; (4) studies that did not provide sufficient data for meta-analysis. Two independent reviewers completed the study selection with disagreement resolved by consensus.

### Data extraction

Three reviewers independently reviewed all included studies and extracted the following data: author, year of publication, region of the dataset, ethnicity of the study population, type of cardiomyopathy, genotype, genotyping method, sample size of the case and control groups, and whether the control group was validated with Hardy-Weinberg principle. Two independent reviewers completed the data extraction with disagreement resolved by consensus.

### Quality assessment

Three reviewers independently rated the quality of the included case-control studies using the Newcastle-Ottawa Scale (NOS). It evaluates studies by means of three major blocks of eight items, specifically study population selection (selection), comparability (comparability), and exposure (exposure) evaluations. The NOS evaluates the quality of the literature using the semi-quantitative principle of the star system, with a total score of 9 stars [23,24]. Two independent reviewers completed the quality assessment with disagreement resolved by consensus.

### Statistical methods

Data were analyzed using STATA version 15.1. The Cochran Q test and *I*^*2*^ statistics were employed to analyze study heterogeneity, and *I*^*2*^ was calculated using the equation: *I*^*2*^ = 100% (Q df)/Q. I-square less than 50% was considered as low heterogeneity between studies and greater than 50% was considered as high heterogeneity [25,26]. Sources of heterogeneity were explored by subgroup analysis. The significance of the combined odds ratio (OR) was determined by the Z-test, in which P ≤ 0.05 was considered significant.

## Results

### Basic characteristics of included studies

**Fig 1:**
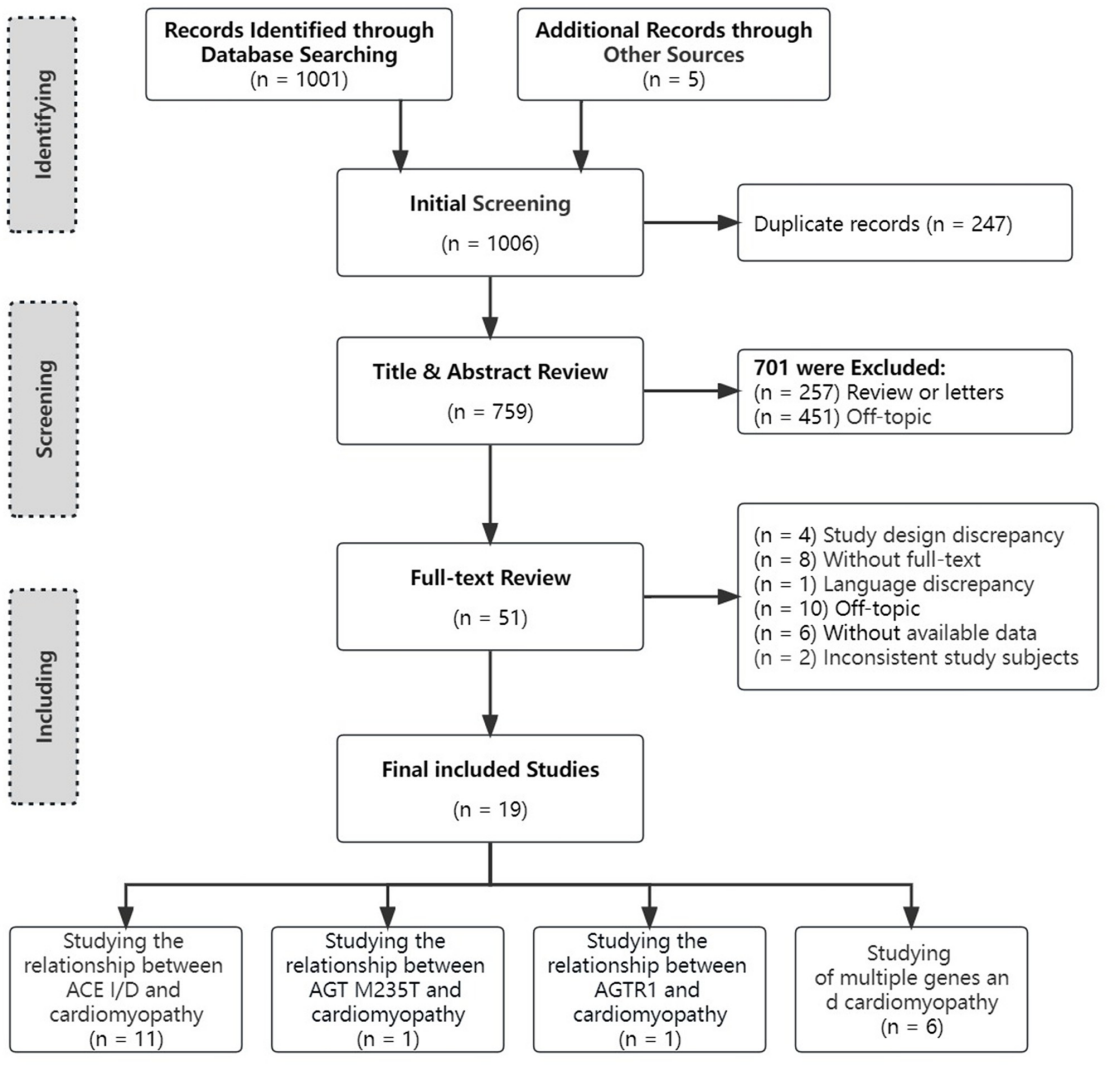
Flow diagram for study selection.

There were 1,006 articles found to be potentially eligible after a thorough literature search. Fifty-one articles were included in the analysis after excluding duplicates and checking the titles and abstracts. Finally, a total of 19 studies [20,27–44] met the inclusion requirements after reading the full text (Fig 1). Due to the multiple research questions included in one study, 30 groups of data were extracted from the 19 studies for meta-analysis.

The main characteristics of the included studies are shown in Table 1. The 19 studies encompass 4,052 patients with cardiomyopathy. Among these participants, 1,554 individuals were diagnosed with HCM, while 2,498 individuals were diagnosed with DCM. Additionally, we included a control group consisting of 5,592 individuals.

**Table 1:** Main characteristics of studies included in the present meta-analysis.

Study	Study area	Type of cardiomyopathy	Confirmation of diagnosis	Genotyping methods	Hardy–Weinberg	Case			Control		
ACE I/D						DD	ID	II	DD	ID	II
Yoneya [44], 1995	Japan	HCM	Echocardiography	PCR	No	13	41	26	17	28	43
Candy [27], 1999	South Africa	DCM	Echocardiography	PCR	Yes	72	72	27	75	71	25
Sanderson [45], 1996	China	DCM	based on the criteria set by the World Health Organization for dilated cardiomyopathy, with left ventricular ejection fraction <40% or fractional shortening <25% on echocardiography	PCR	Yes	12	49	39	13	48	39
Vancura [29], 1999	Czech Republic	DCM	Echocardiography	Unclear	Yes	30	33	27	71	146	70
Tiago [46], 2002	South Africa	DCM	Echocardiography	PCR	No	71	60	26	102	105	18
Montgomery [47], 1995	USA	DCM	All patients had left ventricular dilation (enddiastolic diameter >2.7 cm/m2) (11) and impaired systolic contraction (left ventricular ejection fraction <40% or fractional shortening <25%). Patients with ->50% obstruction of one or more coronary arteries, active myocarditis	PCR	Yes	31	50	18	112	168	84
Tiret [48], 2000	France	DCM	based on impaired left ventricular systolic function (ejection fraction ≤40% on ventriculography, radionucleotide angiography or echocardiography) and left ventricular dilation (end-diastolic volume >140 ml/m2on ventriculography or end-diastolic diameter >34 mm/m2on echocardiography), confirmed over a six-month period.	Unclear	Yes	128	200	94	126	190	71
Yamada [49], 1997	Japan	DCM	based on patient history, physical examination, electrocardiogram, chest x-ray, echocardiography, left ventriculography, and coronary angiography.	PCR	Yes	17	35	36	17	55	50
Yamada [49], 1997	Japan	HCM	based on patient history, physical examination, electrocardiogram, chest x-ray, echocardiography, left ventriculography, and coronary angiography.	PCR	Yes	8	32	31	17	55	50
Berg [33], 2012	Russia	DCM	who had their pathologies, diagnosed in compliance with WHO classification criteria, EF<45%	PCR	Yes	8	9	10	9	41	32
Coto [50], 2010	Spain	HCM	Echocardiography	PCR	No	72	100	35	119	135	46
Yaqoob [51], 2018	Kashmiri	DCM	diagnosed by clinical (Presentation of heart failure in last month of pregnancy or first 5 months postpartum) and echocardiographic (left ventricular ejection fraction <0.45 or M-mode fractional shortening <30% (or both) and end-diastolic dimension >2.7 cm/m2) criteria	PCR	No	11	18	16	3	19	48
Mahjoub [52], 2010	Tunisia	DCM	DCM is diagnosed in the presence of (i) fractional shortening less (LVFS) than 25% and/or ejection fraction (LVFE) less than 45%; (ii) left ventricular end diastolic diameter (LVEDD) greater than 69 mm excluding any known cause of myocardial disease.	PCR	Yes	26	38	12	22	83	4
Rai [53], 2008	Indian	DCM	Echocardiography	PCR	Yes	10	33	8	30	87	47
Rai [53], 2008	Indian	HCM	Echocardiography	PCR	Yes	44	63	11	30	87	47
Deshmukh [38], 2004	USA	DCM	Echocardiography	PCR	No	10	10	0	9	6	6
Fernández-Solà [54], 2002	Spain	DCM	Echocardiography	PCR	No	17	10	3	2	16	9
Biswas [40], 2019	India	HCM	Echocardiography	PCR	Yes	8	35	16	12	52	38
Rani [55], 2017	India	HCM	Echocardiography	PCR	No	27	89	16	42	86	72
Rani [55], 2017	India	DCM	Echocardiography	PCR	No	42	120	15	42	86	72
AGT M235T						TT	MT	MM	TT	MT	MM
Tiago [46], 2002	South Africa	DCM	Echocardiography	PCR	No	102	55	0	167	58	0
Tiret [48], 2000	France	DCM	Based on impaired left ventricular systolic function (ejection fraction ≤40% on ventriculography, radionucleotide angiography or echocardiography) and left ventricular dilation (end-diastolic volume >140 ml/m2on ventriculography or end-diastolic diameter >34 mm/m2on echocardiography), confirmed over a six-month period.	Unclear	Yes	333	88	7	295	93	10
Coto [50], 2010	Spain	HCM	Echocardiography	PCR	No	41	100	64	60	145	95
Kawaguchi [42], 2003	Japan	HCM	Echocardiography	PCR	No	67	28	1	94	61	5
Rani [55], 2017	India	HCM	Echocardiography	PCR	No	43	72	16	20	126	26
Rani [55], 2017	India	DCM	Echocardiography	PCR	No	55	97	11	20	126	26
AGTR1 A1166C						AA	CA	CC	AA	CA	CC
Ishanov [56], 1998	Japan	HCM	Echocardiography	PCR	Yes	88	8	0	139	18	3
Coto [50], 2010	Spain	HCM	Echocardiography	PCR	No	84	94	27	156	114	30
Rani [55], 2017	India	HCM	Echocardiography	PCR	No	2	23	129	0	70	130
Rani [55], 2017	India	DCM	Echocardiography	PCR	No	0	41	156	0	70	130

Abbreviations: HCM, hypertrophic cardiomyopathy; DCM, dilated cardiomyopathy; HW, Hardy-Weinberg principle validation was used for the control group; PCR, polymerase chain reaction.

In all of the 19 studies, echocardiography was used as the diagnostic tool for cardiomyopathy. The included studies were published between 1995 and 2019, covering a wide time range of research in this field. Regarding the method of genetic testing, 17 studies [20,27,28,30,31,33–44] employed the polymerase chain reaction (PCR) technique, while methods were unclear in two studies [29,32].

### Association of ACE I/D genes polymorphism with the risk of cardiomyopathy

**Fig 2:**
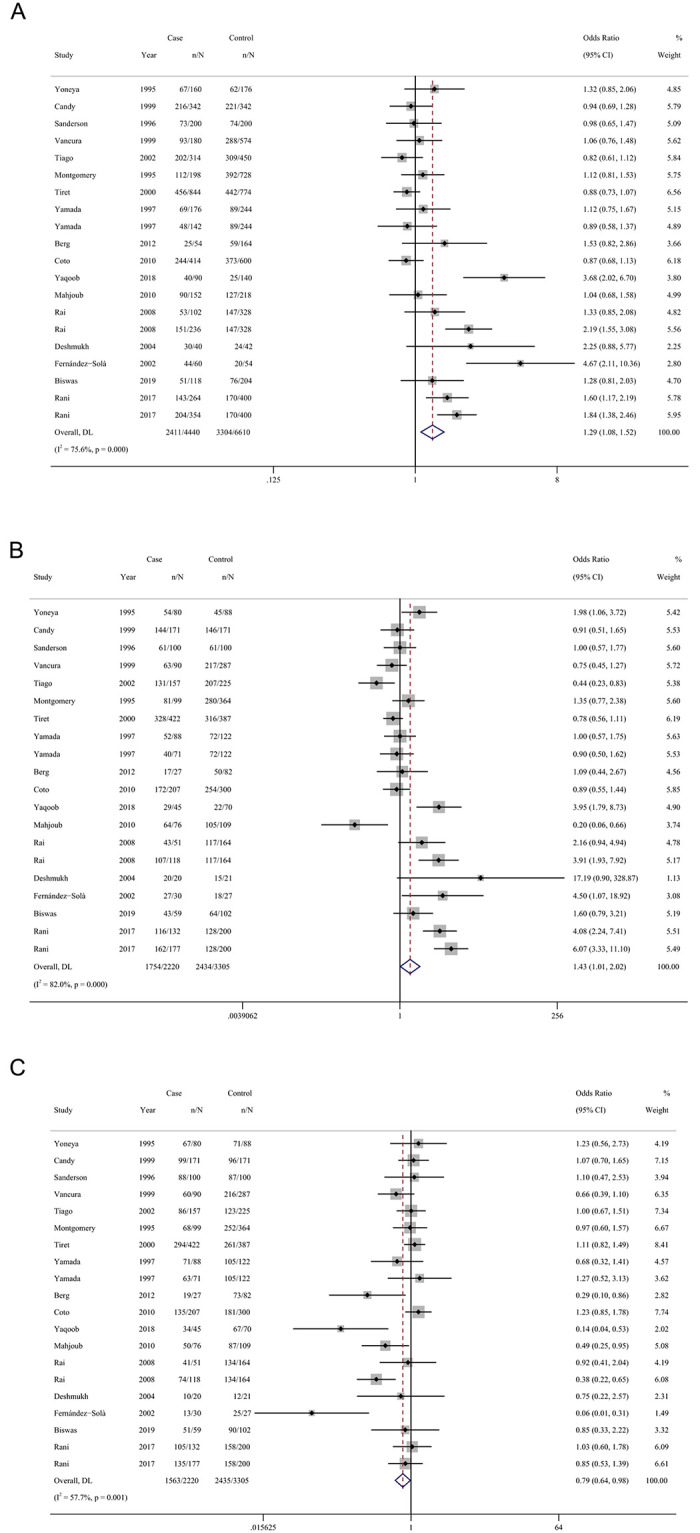
Forest plot for the association between cardiomyopathy and ACE I/D polymorphism. (A) Allelic model, (B) Dominant model, (C) Recessive model.

Twenty studies explored the association between ACE I/D gene polymorphism and the risk of cardiomyopathy. ACE I/D polymorphisms were found to be associated with cardiomyopathy under all three models. The summary OR for the D allele versus I is presented in Fig 2A, with a pooled OR of 1.29 (95 CI% = 1.08–1.52). Under the dominant model (DD+ID vs II), the pooled OR was 1.43 (95 CI% = 1.01–2.02, Fig 2B). Conversely, under recessive model (DD vs ID+II), the pooled OR is 0.79 (95 CI% = 0.64–0.98, Fig 2C).

A subgroup analysis was conducted based on the type of cardiomyopathy (HCM and DCM) and whether the control group used Hardy-Weinberg validation (yes and no). The result is presented in Table 2. Regarding the type of cardiomyopathy, an association was observed between ACE I/D variants and HCM risk under the dominant model, with a pooled OR of 1.820 (DD + ID vs. II: 95% CI = 1.040–3.183; P = 0.036). Additionally, associations were detected between ACE I/D variants and DCM risk under the allelic and recessive models, with pooled ORs of 1.287 (D vs I: 95% CI = 1.043–1.588; P = 0.019), 0.746 (DD vs ID + II: 95% CI = 0.577–0.965; P = 0.025), respectively. In studies validated with Hardy-Weinberg principle, there was an association between ACE I/D variants and cardiomyopathy risk under the recessive model, with a pooled OR of 0.779 (DD vs ID+II: 95% CI = 0.607–1.000; P = 0.050). In studies validated without Hardy-Weinberg principle, there was an association between ACE I/D variants and risk of cardiomyopathy with pooled ORs of 1.647 under the allelic model (D vs I: 95% CI = 1.138–2.384; P = 0.008) and 2.433 under dominant model (DD + ID vs II: 95% CI = 1.151–5.142; P = 0.020). Heterogeneity between studies across subgroups was not eliminated.

**Table 2:** Subgroup analysis of the different genetic models by type of cardiomyopathy and whether controls used the Hardy-Weinberg principle.

Subgroup	Genetic model	Analysis model	Heterogeneity		OR	
			I^2^	P	Overall (95CI%)	P
ACE I/D polymorphism						
HCM	D vs I	Random	77.8%	0.001	1.292 (0.945, 1.767)	0.109
	DD+ID vs II	Random	80.2%	0.001	1.820 (1.040, 3.183)	0.036*
	DD vs ID+II	Random	64.3%	0.016	0.913 (0.594, 1.403)	0.677
DCM	D vs I	Random	75.9%	0.001	1.287 (1.043, 1.588)	0.019*
	DD+ID vs II	Random	82.2%	0.001	1.276 (0.828, 1.966)	0.269
	DD vs ID+II	Random	57.6%	0.004	0.746 (0.577, 0.965)	0.025*
HW	D vs I	Random	55.5%	0.01	1.133 (0.966, 1.328)	0.124
	DD+ID vs II	Random	64.1%	0.001	1.087 (0.807, 1.465)	0.583
	DD vs ID+II	Random	50.2%	0.024	0.779 (0.607, 1.000)	0.050*
NHW	D vs I	Random	85.4%	0.001	1.647 (1.138, 2.384)	0.008*
	DD+ID vs II	Random	87.8%	0.001	2.433 (1.151, 5.142)	0.020*
	DD vs ID+II	Random	68.0%	0.003	0.780 (0.517, 1.178)	0.238
AGT M235T polymorphism						
HCM	T vs M	Random	66.9%	0.049	1.328 (0.944, 1.868)	0.103
	TT+MT vs MM	Fixed	0.0%	0.547	1.115 (0.805, 1.546)	0.513
	TT vs MT+MM	Random	83.4%	0.002	0.561 (0.267, 1.176)	0.126
DCM	T vs M	Random	86.3%	0.001	1.183 (0.708, 1.977)	0.520
	TT+MT vs MM	Fixed	0.0%	0.459	2.090 (1.162, 3.759)	0.014*
	TT vs MT+MM	Random	91.6%	0.001	0.703 (0.296, 1.672)	0.426
HW	T vs M	Random	/	/	1.223 (0.918, 1.630)	0.169
	TT+MT vs MM	Fixed	/	/	1.550 (0.584, 4.112)	0.379
	TT vs MT+MM	Random	/	/	0.817 (0.593, 1.125)	0.216
NHW	T vs M	Random	80.6%	0.001	1.271 (0.901, 1.794)	0.172
	TT+MT vs MM	Fixed	39.5%	0.175	1.280 (0.952, 1.722)	0.103
	TT vs MT+MM	Random	89.2%	0.001	0.592 (0.296, 1.184)	0.139
AGTR1 A1166C polymorphism						
HCM	A vs C	Random	77.3%	0.012	0.770 (0.430, 1.379)	0.379
	AA+CA vs CC	Fixed	62.6%	0.069	0.523 (0.363, 0.753)	0.001*
	AA vs CA+CC	Random	66.7%	0.05	0.886 (0.341, 2.298)	0.803
DCM	A vs C	Random	/	/	0.548 (0.362, 0.828)	0.004*
	AA+CA vs CC	Fixed	/	/	0.488 0.311, 0.766)	0.002*
	AA vs CA+CC	Random	/	/	/	/
HW	A vs C	Random	/	/	1.865 (0.820, 4.239)	0.137
	AA+CA vs CC	Fixed	/	/	4.289 (0.219, 83.934)	0.001*
	AA vs CA+CC	Random	/	/	0.602 (0.255, 1.418)	0.245
NHW	A vs C	Random	39.7%	0.191	0.595 (0.451, 0.785)	0.001*
	AA+CA vs CC	Fixed	41.0%	0.184	0.492 (0.369, 0.655)	0.001*
	AA vs CA+CC	Random	54.9%	0.137	0.812 (0.105, 6.308)	0.842

Abbreviations: HCM, hypertrophic cardiomyopathy; DCM, dilated cardiomyopathy; HW, Hardy-Weinberg principle validation was used for the control group; NHW, The control group did not use Hardy-Weinberg principle validation; OR, Odds ratio; CI, Confidence interval;

*, *P* ≤0.05.

### Association of AGT M235T genes polymorphism with the risk of cardiomyopathy

**Fig 3:**
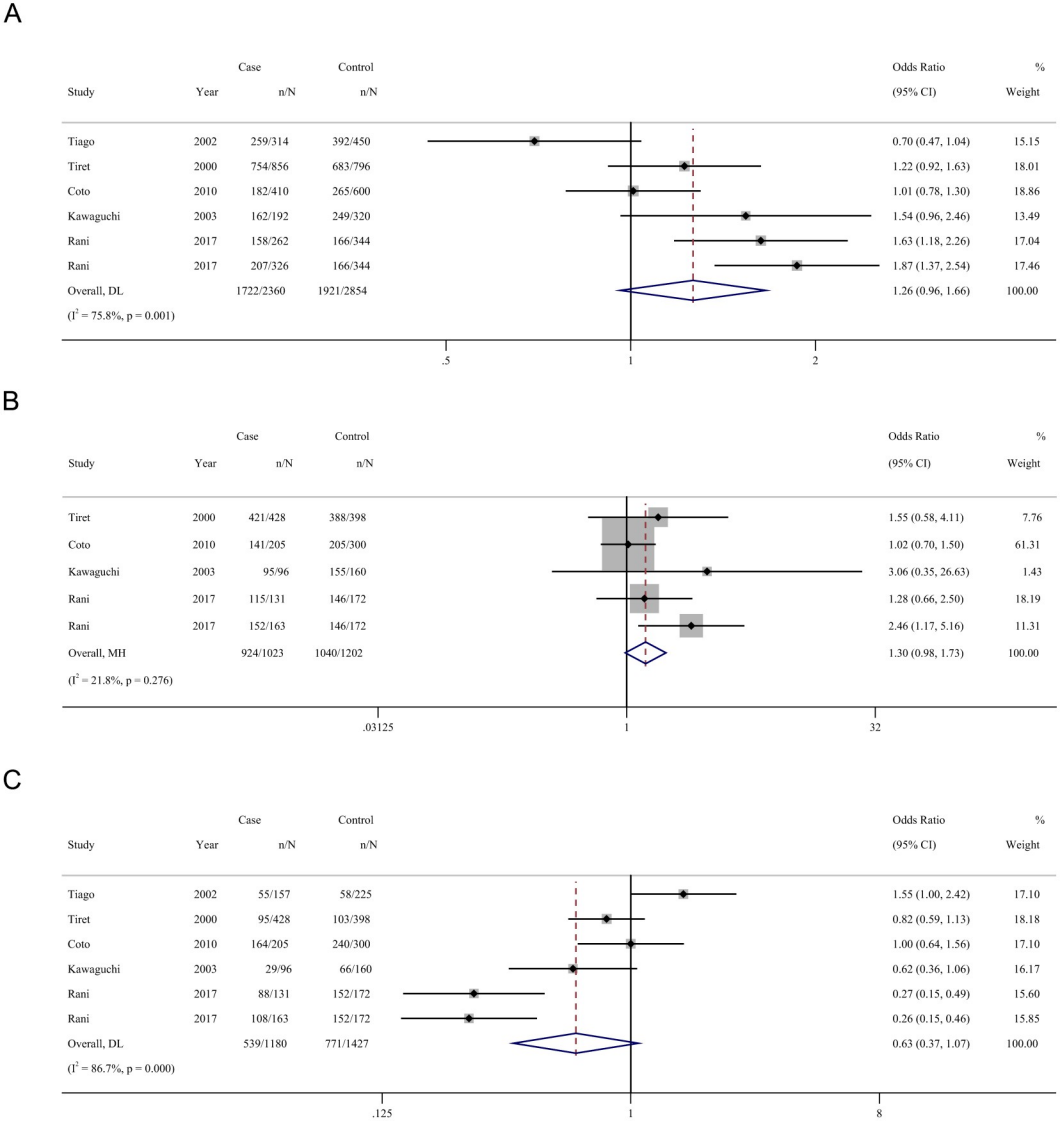
Forest plot for the association between cardiomyopathy and AGT M235T polymorphism. (A) Allelic model, (B) Dominant model, (C) Recessive model.

Five studies have been conducted to investigate the relationship between AGT M235T polymorphism and the risk of cardiomyopathy. AGT M235T polymorphism and cardiomyopathy were not significantly correlated. Under the allelic model T vs M, a pooled OR of 1.26 (95 CI% = 0.96–1.66) is observed (Fig 3A). Under the dominant model TT+MT vs MM, the pooled OR is 1.30 (95 CI% = 0.98–1.73, Fig 3B), while under the recessive model TT vs MT+MM, the pooled OR is 0.63 (95 CI% = 0.37–1.07, Fig 3C).

In the subgroup analysis, an association was detected between the AGT M235T polymorphism and DCM risk under the dominant model with a pooled OR of 2.090 (TT+MT vs MM: 95% CI = 1.162–3.759; P = 0.014). No association was found between the remaining subgroups of AGT M235T (Table 2). Inter-study heterogeneity was eliminated in the HCM, DCM, and NHW groups (TT+MT vs MM: P heterogeneity = 0.547, I2 = 0.0%; TT+MT vs MM: *P*_*heterogeneity*_ = 0.459, I2 = 0.0%; TT+MT vs MM: *P*_*heterogeneity*_ = 0.175, I2 = 39.5%).

### Association of AGTR1 A1166C genes polymorphism with the risk of cardiomyopathy

**Fig 4:**
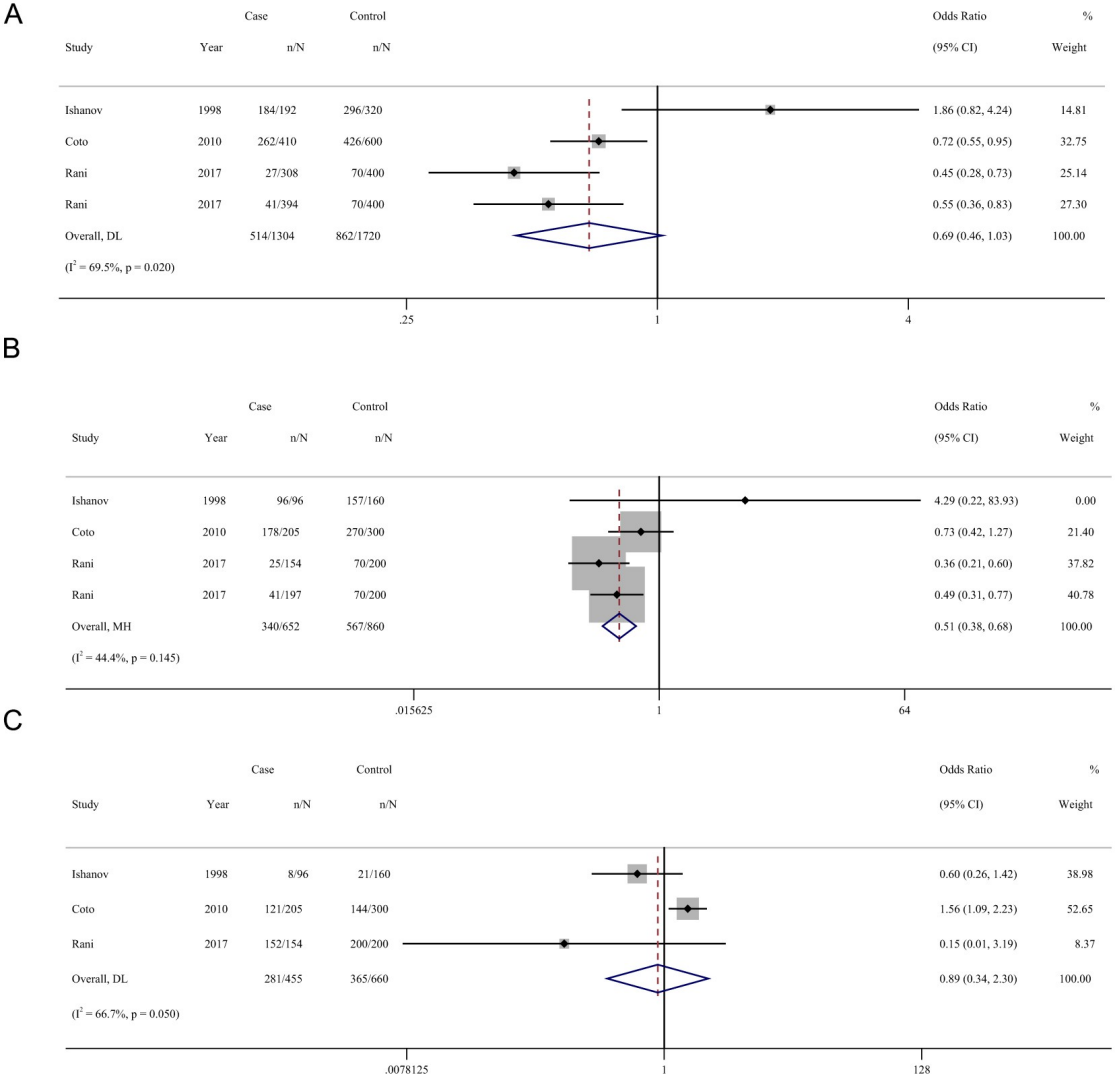
Forest plot for the association between cardiomyopathy and AGTR1 A1166C polymorphism. (A) Allelic model, (B) Dominant model, (C) Recessive model.

Among the four studies included in our meta-analysis that investigated the association between AGTR1 A1166C polymorphisms and the risk of cardiomyopathy, no significant association was observed when analyzing the allelic model A vs C (OR = 0.69, 95% CI = 0.46–1.03, Fig 4A) and the recessive model AA vs CA+CC (OR = 0.89, 95% CI = 0.34–2.30, Fig 4C). However, a significant association was found under the dominant model AA+CA vs CC (OR = 0.51, 95% CI = 0.38–0.68, Fig 4B).

The results of subgroup analysis are shown in Table 2. An association between AGTR1 A1166C polymorphism and HCM risk was detected under the dominant model with a pooled OR of 0.523 (AA+CA vs CC: 95% CI = 0.363–0.753; P = 0.001). Associations were detected between AGTR1 polymorphisms and DCM risk under the allelic model and dominant model, with pooled ORs of 0.548 (A vs C: 95% CI = 0.362–0.828; P = 0.004) and 0.488 (AA+CA vs CC: 95% CI = 0.311–0.766; P = 0.002), respectively. In studies validated with Hardy-Weinberg principle, there was an association between AGTR1 polymorphism and cardiomyopathy risk under the dominant model with a pooled OR of 4.289 (AA+CA vs CC: 95% CI = 0.219–83.934; P = 0.001). In studies validated without Hardy-Weinberg principle, there was an association between AGTR1 polymorphism and risk of cardiomyopathy with pooled ORs of 0.595 (allelic model, A vs C: 95% CI = 0.451–0.785; P = 0.008) and 0.492 (dominant model, AA+CA vs CC: 95% CI = 0.369–0.1655; P = 0.001). Heterogeneity between studies was not eliminated except in the NHW group (A vs C: *P*_*heterogeneity*_ = 0.191, I^2^ = 39.7%; AA+CA vs CC: *P*_*heterogeneity*_ = 0.184, I^2^ = 41.0%).

### Quality assessment

Newcastle-Ottawa Scale (NOS) scores for the included studies are shown in S2 Table. All studies included consecutive or representative series of cases, and all studies controlled for the most important confounding factors. Only a small number of studies did not describe the control group in question. Twenty-nine studies were of good quality, with a quality assessment score of 7 or more (out of 9).

### Publication bias

The funnel plots for all the above studies are presented in S1 Fig. No publication bias was observed, as the shape of the funnel plots did not seem to show significant asymmetry in each meta-analysis. This was further verified by Egger’s test (ACE D/I: P = 0.149, AGT M235T: *P* = 0.149, AGTR1 A1166C: *P* = 0.349).

## Discussion

In this meta-analysis, we reviewed all eligible studies that evaluate the associations between ACE I/D, AGT M235T, and AGTR1 A1166C gene polymorphisms and the risk of cardiomyopathy. The ACE I/D gene polymorphisms were found to be associated with cardiomyopathy, while M235T and AGTR1 A1166C gene polymorphisms and cardiomyopathy were not significantly correlated.

ACE plays a crucial role in the synthesis of angiotensin II (Ang II), which in turn triggers various cellular processes such as proliferation, migration, hypertrophy, as well as the upregulation of proinflammatory cytokines and matrix metalloproteinases. Consequently, the overexpression of Ang II has been implicated in the development of cardiomyopathy. In the present study, ACE I/D gene polymorphism was found to be associated with an increased risk of DCM and HCM, which is consistent with previous meta-analysis. Yuan et al. conducted a meta-analysis comprising 2,972 participates and found that D allele carrier is a risk allele, which indicates that ACE gene polymorphism may be a genetic risk factor for HCM [13]. Luo conducted a meta-analysis comparing the DI/II genotype to DD genotype and found an OR of 0.73, which indicated the potential association between ACE I/D gene polymorphism and HCM [57]. In a meta-analysis conducted by Shen et al. [58], ACE I/D gene polymorphism was associated with the increased risk of DCM/HCM in Asians, but not in Caucasians. In a meta-analysis conducted by Yang, ACE I/D polymorphism may be associated with HCM but not DCM susceptibility [59].

Extensive attention has been devoted to investigating the potential link between the AGT M235T polymorphism and the development of cardiomyopathy. Previous studies have yielded divergent outcomes. Kawaguchi’s study identified the gene polymorphism of M235T as a predisposing factor for cardiac hypertrophy in HCM [42]. Likewise, Ranni et al. [55] reported an association between the M235T polymorphism and DCM, HCM and RCM. In contrast, Tiago et al. [46] observed no correlation between the M235T polymorphism and DCM in Black South Africans. Tiret et al. [48] explored a cohort of 428 participants with DCM and found no link between the M235T polymorphism and either the risk or severity of DCM. Similarly, Coto et al. [50] did not identify an association between M235T and HCM. Upon pooling the results, our analysis revealed no significant correlation between AGT M235T polymorphism and cardiomyopathy. However, subgroup analysis identified that the AGT M235T polymorphism is association with increased DCM risk in the dominant model, which indicates that the presence of one or two copies of T allele significantly increases the risk of DCM. It has been reported that elevated levels of serum AGT were observed in the heart and kidney of individuals who possess the 235T allele [60]. AGT plays a pivotal role in the production of angiotensin II through its interaction with renin [61]. Angiotensin II, in turn, has been demonstrated to promote vasoconstriction, increases blood pressure, and stimulates the release of aldosterone that may ultimately lead to the development of DCM. While the AGT M235T gene polymorphism is associated with an increased DCM risk, it remains unclear whether this genetic variant directly contributes to the pathogenesis of DCM or if it serves as a marker for other genetic or environmental factors. Additional research, including functional studies and animal models, is needed to establish a causal link and to uncover the precise mechanisms by which this gene polymorphism influences DCM susceptibility.

A noteworthy observation was made regarding the increased frequency of AGTR1 A1166C carriers among the HCM patients without sarcomeric mutations, implying a potential risk associated with the AGTR1 A1166C polymorphism in HCM [50]. Similarly, Rani’s study reported a higher prevalence of AGTR1 1166CC in both HCM and DCM cases [55]. However, a study conducted in a Japanese cohort failed to establish an association between AGTR1 A1166C gene polymorphisms and HCM [56]. Our comprehensive analysis, encompassing all available data, did not reveal a significant correlation between the AGTR1 A1166C polymorphism and cardiomyopathy. Nevertheless, our findings support an association between the AGTR1 A1166C gene polymorphism and HCM risk under the dominant model, as well as an association with DCM under the allelic and dominant models. Previous meta-analyses confirmed the association of the AGTR1 A1166C polymorphism with coronary heart disease [62,63] and essential hypertension [64]. However, only limited studies have investigated its relationship with cardiomyopathy. To the best of our knowledge, this is the first meta-analysis examining the association between AGTR1 A1166C and cardiomyopathy. Further high-quality and large-scale trials are warranted to shed more light on this topic.

It is important to interpret the results of our meta-analysis with caution due to several limitations. Firstly, heterogeneity is a common issue encountered in meta-analyses of association studies and it was also observed in our study, which could influence the results. The heterogeneity may arise from several factors, including variations in study designs, or differences in sample selection among the included studies. Secondly, previous literature has reported that the association between ACE D/I polymorphism and M235T polymorphism with cardiomyopathy may vary among different ethnicities. However, we were unable to perform subgroup analyses based on different ethnicities due to the limited number of studies available for each specific ethnicity. Thirdly, gene-gene interactions may also contribute to the development of cardiomyopathy. However, limited information was included in the original studies. These limitations highlight the need for further research to explore the potential impact of different ethnicities and gene-environment interactions on the association between ACE D/I, AGTR1 A1166C, and M235T polymorphisms with cardiomyopathy.

## Conclusion

The current meta-analysis suggests that polymorphisms in the ACE I/D may be a genetic risk factor for cardiomyopathy. The dominant model detected associations between ACE I/D polymorphisms and HCM risk, whereas the allelic and recessive models detected associations between ACE I/D polymorphisms and DCM risk. Under the dominant hypothesis, an association between AGT M235T polymorphism and DCM risk was discovered. Furthermore, a relationship was discovered between AGTR1 polymorphism and HCM risk under the dominant model, as well as an association between AGTR1 polymorphism and DCM risk under the allelic model and the dominant model.

## Supporting information

S1 Checklist PRISMA 2020 checklist. (DOCX)

S1 Table Search strategies used in this study. (DOCX)

S2 Table Results of quality assessment using the Newcastle-Ottawa Scale for observational studies. (DOCX)

S1 Fig Publication bias for ACE I/D (A), AGT M235T (B), and AGTR1 A1166C (C). (TIF)
