# Supplementary material for: Impact of the gene polymorphisms in the renin-angiotensin system on cardiomyopathy risk: A meta-analysis
Source: PLoS One. 2024 Jan 2;19(1):e0295626. doi: 10.1371/journal.pone.0295626 (PMC10760857; doi:10.1371/journal.pone.0295626)
Supplement: S1 Table — (DOCX) [file pone.0295626.s002.docx]

S1 Table. Search strategies used in this study

| **Database** | **Search strategy** |
| --- | --- |
| **Pubmed** | #1 "Cardiomyopathies"[Mesh]  #2 "Cardiomyopathies"[Title/Abstract] OR "Cardiomyopathy"[Title/Abstract] OR "DCM"[Title/Abstract] OR "HCM"[Title/Abstract] OR "RCM"[Title/Abstract]  #3 #1 OR #2  #4 "Angiotensin-Converting Enzyme"[Title/Abstract] OR "ACE"[Title/Abstract] OR "ACE I/D"[Title/Abstract] OR "rs4646994"[Title/Abstract] OR "AGT M235T"[Title/Abstract] OR "angiotensinogen angiotensin II Type 1 receptor"[Title/Abstract] OR "AGTR1"[Title/Abstract] OR "a1166c"[Title/Abstract]  #5 "Genetic Variants"[Title/Abstract] OR "polymorphism"[Title/Abstract] OR "susceptibility"[Title/Abstract] OR "genetic association study"[Title/Abstract]  #6 #3 AND #4 AND #5 |
| **Embase** | #1 'Cardiomyopathies'/exp  #2 'Cardiomyopathy' OR 'DCM' OR 'HCM' OR 'RCM'  #3 #1 OR #2  #4 'Angiotensin-Converting Enzyme' OR 'ACE' OR 'ACE I/D' OR 'rs4646994' OR 'AGT M235T' OR 'angiotensinogen angiotensin II Type 1 receptor' OR 'AGTR1' OR 'a1166c'  #5 'Genetic Variants' OR 'polymorphism' OR 'susceptibility' OR 'genetic association study'  #6 #3 AND #4 AND #5 |
| **Web of Science** | #1 TI=("Cardiomyopathy" OR "Cardiomyopathies" OR "Myocardiopathies" OR "Myocardiopathy" OR "Myocardial" OR "DCM" OR "HCM" OR "RCM") OR AB=("Cardiomyopathy" OR "Cardiomyopathies" OR "Myocardiopathies" OR "Myocardiopathy" OR "Myocardial" OR "DCM" OR "HCM" OR "RCM")  #2 TI=("Angiotensin-Converting Enzyme" OR "ACE" OR "ACE I/D" OR "rs4645994" OR "AGT M235T" OR "angiotensinogen angiotensin II Type 1 receptor" OR "AGTR1" OR "a1166c") OR AB=("Angiotensin-Converting Enzyme" OR "ACE" OR "ACE I/D" OR "rs4646994" OR "AGT M235T" OR "angiotensinogen angiotensin II Type 1 receptor" OR "AGTR1" OR "a1166c")  #3 TI=("Genetic Variants" OR "polymorphism" OR "susceptibility" OR "genetic association study") OR AB=("Genetic Variants" OR "polymorphism" OR "susceptibility" OR "genetic association study")  #4 #1 AND #2 AND #3 |
| **Cochrane library** | #1 [mh "Cardiomyopathies"]  #2 "Cardiomyopathies" OR "Cardiomyopathy" OR "DCM" OR "HCM" OR "RCM"  #3 #1 OR #2  #4 "Angiotensin-Converting Enzyme" OR "ACE" OR "ACE I/D" OR "rs4646994" OR "AGT M235T" OR "angiotensinogen angiotensin II Type 1 receptor" OR "AGTR1" OR "a1166c"  #5 "Genetic Variants" OR "polymorphism" OR "susceptibility" OR "genetic association study"  #6 #3 AND #4 AND #5 |
