# Supplementary material for: Impact of the gene polymorphisms in the renin-angiotensin system on cardiomyopathy risk: A meta-analysis
Source: PLoS One. 2024 Jan 2;19(1):e0295626. doi: 10.1371/journal.pone.0295626 (PMC10760857; doi:10.1371/journal.pone.0295626)
Supplement: S2 Table — (DOCX) [file pone.0295626.s003.docx]

S2 Table. Results of quality assessment using the Newcastle-Ottawa Scale for observational studies.

| **Author** | **Year** | **SELECTION** | | | | **COMPARABILITY** | **EXPOSURE** | | | **Score** |
| --- | --- | --- | --- | --- | --- | --- | --- | --- | --- | --- |
|  |  | Case Definition | Representativeness | Selection of Controls | Definition of Controls | Comparability of Cases and Control | Ascertainment of Exposure | Same methods? | Non-Response Rate |  |
| Yoneya | 1995 | ★ | ★ | ★ | ★ | ★ | ★ | ★ | ★ | 8 |
| Candy | 1999 | ★ | ★ | ★ | ★ | ★ | ★ | ★ | ★ | 8 |
| Sanderson | 1996 | ★ | ★ | ★ | ★ | ★ | ★ | ★ | ★ | 8 |
| Vancura | 1999 | ★ | ★ | ★ | ★ | ★ | ★ | ★ | ★ | 8 |
| Tiago | 2002 | ★ | ★ | ★ | ★ | ★ | ★ | ★ | ★ | 8 |
| Montgomery | 1995 | ★ | ★ | ★ | ★ | ★ | ★ | ★ | ★ | 8 |
| Tiret | 2000 | ★ | ★ | ★ | ★ | ★ | ★ | ★ | ★ | 8 |
| Yamada | 1997 | ★ | ★ | ★ | ★ | ★ | ★ | ★ | ★ | 8 |
| Yamada | 1997 | ★ | ★ | ★ | ★ | ★ | ★ | ★ | ★ | 8 |
| Berg | 2012 | ★ |  |  | ★ | ★ | ★ | ★ | ★ | 6 |
| Coto | 2010 | ★ | ★ | ★ | ★ |  | ★ | ★ | ★ | 7 |
| Yaqoob | 2018 | ★ | ★ | ★ | ★ | ★ | ★ | ★ | ★ | 8 |
| Mahjoub | 2010 | ★ |  |  | ★ | ★ | ★ | ★ | ★ | 6 |
| Rai | 2008 | ★ | ★ | ★ | ★ | ★★ | ★ | ★ | ★ | 9 |
| Rai | 2008 | ★ | ★ | ★ | ★ | ★★ | ★ | ★ | ★ | 9 |
| Deshmukh | 2004 | ★ |  | ★ | ★ | ★ | ★ | ★ | ★ | 7 |
| Fernández-Solà | 2002 | ★ | ★ | ★ | ★ | ★ | ★ | ★ | ★ | 8 |
| Biswas | 2019 | ★ | ★ | ★ | ★ | ★ | ★ | ★ | ★ | 8 |
| Rani | 2017 | ★ | ★ | ★ | ★ | ★★ | ★ | ★ | ★ | 9 |
| Rani | 2017 | ★ | ★ | ★ | ★ | ★★ | ★ | ★ | ★ | 9 |
| Tiago | 2002 | ★ | ★ | ★ | ★ | ★ | ★ | ★ | ★ | 8 |
| Tiret | 2000 | ★ | ★ | ★ | ★ | ★ | ★ | ★ | ★ | 8 |
| Coto | 2010 | ★ | ★ | ★ | ★ |  | ★ | ★ | ★ | 7 |
| Kawaguchi | 2003 | ★ | ★ | ★ | ★ | ★★ | ★ | ★ | ★ | 9 |
| Rani | 2017 | ★ | ★ | ★ | ★ | ★★ | ★ | ★ | ★ | 9 |
| Rani | 2017 | ★ | ★ | ★ | ★ | ★★ | ★ | ★ | ★ | 9 |
| Ishanov | 1998 | ★ | ★ | ★ | ★ | ★★ | ★ | ★ | ★ | 9 |
| Coto | 2010 | ★ | ★ | ★ | ★ |  | ★ | ★ | ★ | 7 |
| Rani | 2017 | ★ | ★ | ★ | ★ | ★★ | ★ | ★ | ★ | 9 |
