# Supplementary figures and images for: Impact of the gene polymorphisms in the renin-angiotensin system on cardiomyopathy risk: A meta-analysis
Source: PLoS One. 2024 Jan 2;19(1):e0295626. doi: 10.1371/journal.pone.0295626 (PMC10760857; doi:10.1371/journal.pone.0295626)

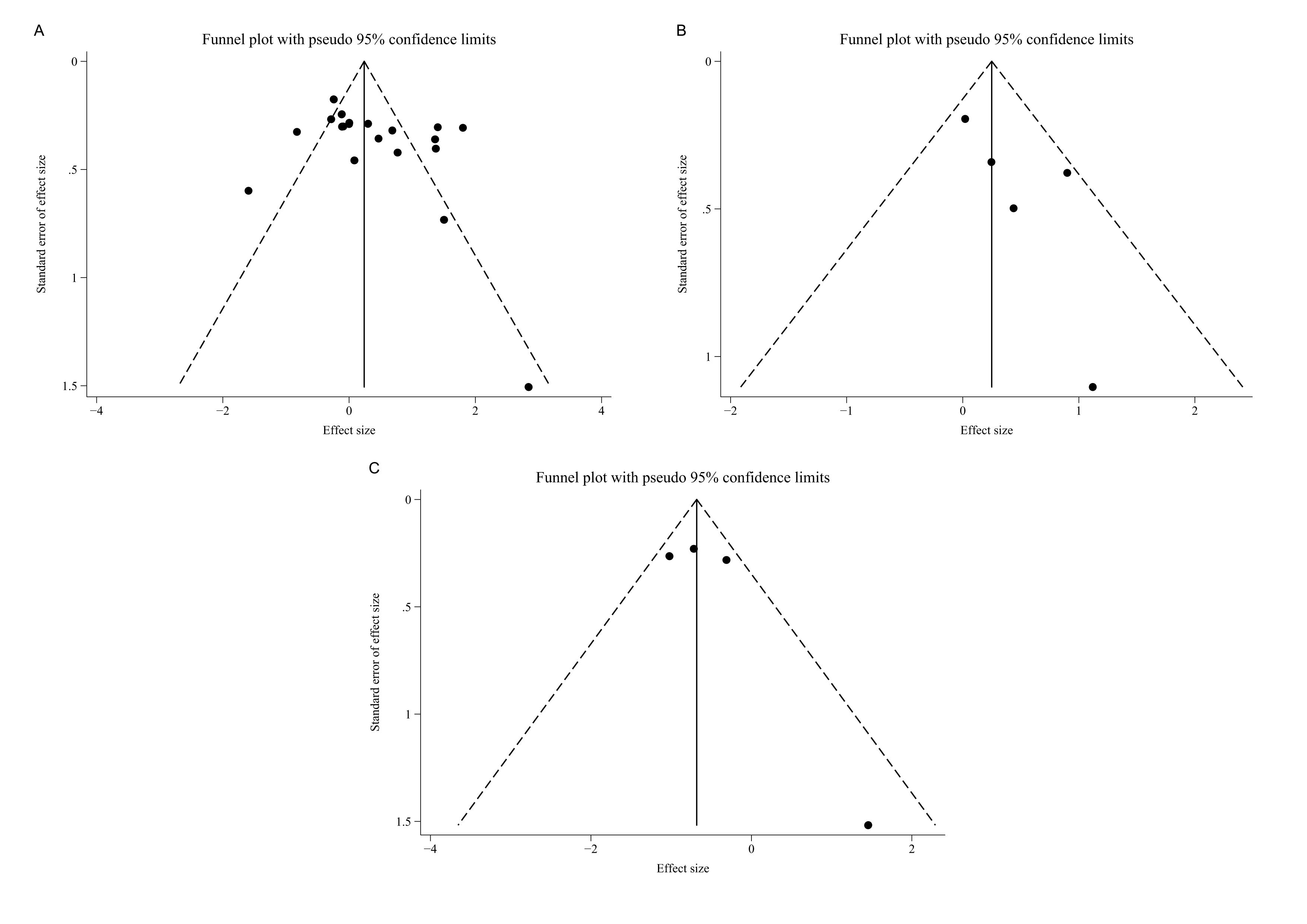

Supplement: S1 Fig — (TIF) [file pone.0295626.s004.tif]
